# Synergistic impacts of organic modifications and foliar phytostimulants on soil properties, growth, yield, and chemical constituents of borage (*borago officinalis* L.)

**DOI:** 10.1186/s12870-025-07430-9

**Published:** 2025-10-02

**Authors:** Mostafa F. Ibrahim, Mohamed A. Mahmoud, Fatma A. Fouad, Ahmed Ali Mahmoud, Mahmoud Ahmed Mohamed, Ahmed A. Abdelrhman, Ahmed M. Ali, Hassan M. Al-Sayed

**Affiliations:** 1 Department of Horticulture, Faculty of Agriculture, Al-Azhar University, Assiut, Egypt; 2 Department of Agronomy (Biochemistry branch, Faculty of Agriculture, Al- Azhar University, Assiut, Egypt; 3 Central laboratory for Organic Farming, Agricultural Research Centre, Giza, Egypt; 4 Department of Soil Fertility and Plant Nutrition, Soil, Water and Environment Research Institute, Agricultural Research Center, 12619 Giza, Egypt; 5 Department of Soils and Water, Faculty of Agriculture, Al-Azhar University, Assiut, 71524 Egypt

**Keywords:** Borage, Compost, Spent mushroom substrate, Phytostimulants

## Abstract

**Background:**

The use of organic waste provides a promising long-term amendment strategy for restoring low soil fertility. The present study aimed to evaluate the impact of organic materials, phytostimulant extracts, and their combined application on soil characteristics, vegetative growth, yield parameters, and chemical composition of borage plants.

**Experimental plan:**

The investigation was performed using a split-plot design with three replicates. The main factor (organic materials) included three treatments: control, compost, and spent mushroom substrate (SMS). The secondary factor comprised five treatments: control (tap water), seaweed extract (SWE) at 2 and 4 g L^−1^, and spirulina extract (SE) at 2 and 4 g L^−1^, applied as foliar treatments. All possible combinations of organic amendments and foliar treatments were applied to evaluate their individual and interactive effects on the studied parameters.

**Result:**

The results demonstrated that foliar application of phytostimulant extracts at different concentrations significantly enhanced plant growth parameters, including height, number of branches, herb biomass, flowering, and yield, while having no observable effect on soil properties. Spent mushroom substrate was more effective than compost in enhancing plant growth, yield, and chemical properties. Across two growing seasons, spent mushroom substrate in combination with spirulina algae extract (4 g L^−1^) increased plant height by 20%, branch number by 28%, and herb wet weight by 46% compared to the control on an average basis of two seasons. Consequently, herb dry weight and seed yield increased by 46% and 52%, respectively, based on the average of both seasons.

**Conclusions:**

The principal component analysis revealed that the combination of compost and spent mushroom substrate with phytostimulants improved soil properties, vegetative growth, flowering, yield, nutrient content, and chemical composition. Organic matter was identified as a key factor influencing the performance of borage plants. Combining organic materials with phytostimulants can significantly improve borage yield in soils with limited organic matter.

## Introduction

 Borage (*Borago officinalis* L.) is an annual herb from the Boraginaceae family. It is native to the Mediterranean region but is now cultivated worldwide due to its medicinal value [1]. The plant typically grows to a height of 30 to 60 cm and is characterized by its coarse, hairy stems and leaves [2]. Its flowers are star-shaped with hues ranging from sapphire blue to violet and white, and flowers are a rich source of vitamin E, providing antioxidant advantages to shield cells from harm [3]. In addition, borage flowers contain sugars such as sucrose, glucose, and fructose, as well as organic acids, most notably succinic acid. They also provide essential minerals that support overall human health, such as calcium and potassium [4]. Furthermore, the flowers contain ash and alkaloids [5, 6]. Borage seeds are a valuable source of essential fatty acids, particularly gamma-linolenic acid (GLA), which accounts for 26–35% of the total fatty acid content. GLA is known for its anti-inflammatory and analgesic properties, helping to relieve pain associated with conditions such as headaches and muscle aches [7, 8]. Additionally, this plant possesses pain-relieving and anti-inflammatory effects, helping to alleviate discomfort associated with conditions such as headaches and muscle aches [4].

Sustainable agriculture aims to maintain high productivity while minimizing the environmental impact of cultivation activities and practices [9, 10]. This approach encourages the use of natural inputs, such as organic amendments and biostimulants, to enhance soil health, crop performance, and long-term ecosystem resilience. Insufficient soil organic matter is a major limiting factor in soil health and agricultural productivity, particularly in arid and semi-arid regions [11]. Compost enhances soil fertility by increasing organic matter levels, enhancing water retention, lowering soil pH, and optimizing soil structure, chemical composition, and nutrient availability [12–15]. Moreover, the nutrient composition of compost can vary depending on the source material, but it generally provides a balanced nutrient supply to plants [16, 17].

Spent mushrooms (Pleurotus ostreatus) are widely cultivated for their nutritional value and ease of growth. However, their production results in large quantities of spent mushroom substrate (SMS), which is often discarded. Recent studies have shown that SMS can be a valuable resource in organic farming, offering with benefits encompassing improved soil structure, enhanced nutrient availability, and increased plant growth [18, 19].

Phytostimulants, also known as plant biostimulants, are a diverse group of natural and synthetic compounds that enhance plant growth, yield [20], and the quality and biochemical characteristics of agricultural products [21, 22]. These compounds include amino acids, various plant hormones, seaweed extracts, humic substances, beneficial microbes, and some inorganic minerals such as iodine (I) and selenium (Se) [23]. Phytostimulants boost stress tolerance, maximize photosynthesis, improve nutrient uptake, and stimulate root growth through a variety of methods [24].

The application of seaweed extracts has been associated with increased fruit set and improved fruit size and quality in citrus plants [25]. Seaweed extract contains various plant growth stimulants, which are primarily classified into three groups of phytohormones: gibberellins, auxins, and cytokinins. In addition to these hormones, seaweed extract typically contains 10% or more potassium salts, amino acids, and other bioactive compounds. These compounds enhance nutrient uptake and movement in plants, thereby boosting health and growth. Additionally, they it enhance blooming and increase a plant’s ability to withstand stress [26].

Spirulina, a blue-green alga, is recognized for its nutrient-dense composition, boasting high amounts of proteins, minerals, vitamins, and essential fatty acids. Its application in agriculture as a biostimulant has gained attention due to its potential to enhance plant growth and improve seed yield. It contains a wide assortment of nutrients that are beneficial for plant growth. When applied to the soil or used as a foliar spray, it can enhance the availability of vital minerals, specifically nitrogen, phosphorus, and potassium. Studies have shown that spirulina can act as a biofertilizer, increasing soil fertility and encouraging healthier plant growth [27].

This study aimed to evaluate the potential effects of different organic amendments, including compost and spent mushroom substrate, and foliar- application of seaweed extracts, on the growth characteristics, yield components, and active constituents of borage grown under deficient organic matter condition.

## Materials and methods

**Fig. 1:**
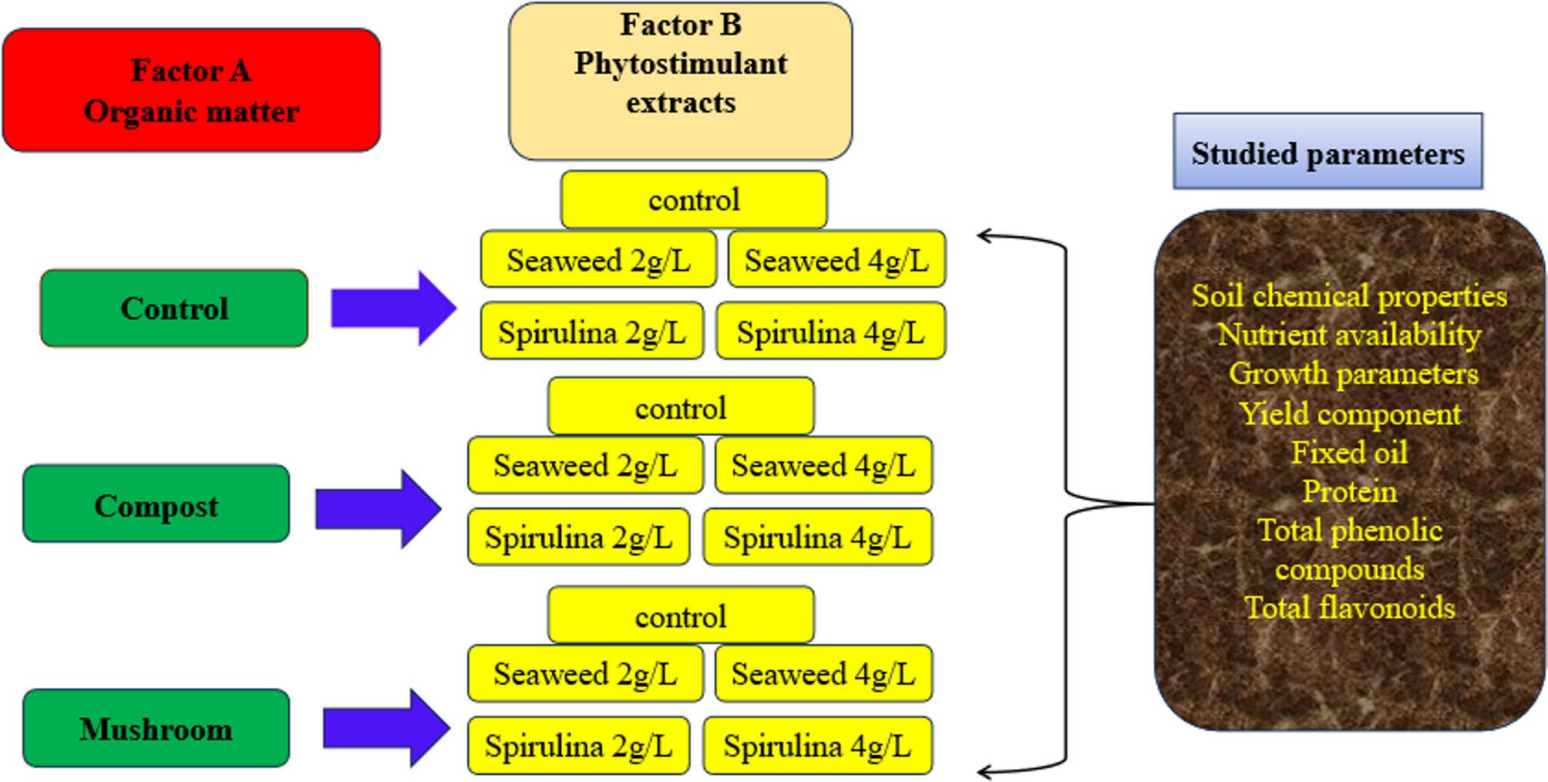
General description of the research paper

This study was carried out at consecutive seasons (2021/2022 and 2022/2023) at the Experimental Farm, College of Agriculture, Al-Azhar University, located in Assiut, Egypt. The experiment employed a split-plot design with three replications. Organic matter served as the main plot factor (A), by three treatments (control, compost at 11.9 t h^−1^, spent mushroom substrate 6.4 t h^−1^), while phytostimulant treatments (subplots, B) were (control, seaweed extract 2 and 4 g L^−1^, and spirolina algae extract 2 and 4 g L^−1^) therefore, the interactions number of 15 treatments (Fig. 1). Borage seeds were purchased from the Medicinal and Aromatic Plant Research Department in El-Qanater El-Khairia, El-Kaliobia, Egypt (. 1.

Borage seeds were planted in nursery beds on October 4th of the two seasons. After 35 days (November 10th), healthy and uniform seedlings were transplanted into the experimental plot. The soil was double plowed orthogonally, followed by leveling and division into experimental units. Each unit (plot) measured 3 m by 1.8 m and contained 3 rows spaced 60 cm apart, with the distance between the plants 40 cm between each other. On 21 days after planting, thinning was performed to maintain one plant per hill. Consistent with local practices, irrigation was applied through immersion once every 25 days. Manual weeding occurred 30 and 60 days after planting (DAS) in both growing seasons, timed before irrigation events. Physical and chemical characteristics were examined according [28] as shown in Table (1).

**Table 1:** Physical and chemical properties of the examined soil before the two successive growing seasons

Studied soil		
Properties	Before 1 st season	Before 2 st season
Sand (%)	20.00	18.30
Silt (%)	56.50	59.60
Clay (%)	23.50	22.10
Texture	Silty Loam	Silty Loam
(pH)	8.12	8.11
EC (dSm^−1^)	1.10	1.12
CaCO_3_ (g kg^−1^)	25.40	25.1
Organic matter (g kg^−1^)	7.30	7.80
Available–N (mg kg^−1^)	35	37
Available–P (mg kg^−1^)	5.81	5.83
Available–K (mg kg^−1^)	275	277

Each value represents a mean of three replicates

Based on information from the Egyptian Ministry of Agriculture and Land Reclamation, superphosphate (15.5% P_2_O_5_) was applied at 75 kg ha^−1^ to each plot during soil plowing. Nitrogen (150 kg ha⁻¹), applied as 33.5% ammonium nitrate, and potassium (125 kg ha⁻¹), applied as potassium sulfate, were administered in two equal splits at 30 and 60 days after sowing (DAS) to support vegetative growth and enhance flowering and yield development in borage plants. Compost, designated as “compost the ‘EL-Neel’ product was procured from the Egyptian Company for Solid Waste Utilization, located in New Minia City, Egypt. The waste of spent mushrooms was brought from a private farm to produce oyster mushroom in Sohag Governorate, Egypt. The chemical analysis of the used compost and mushroom waste is shown in (Tables 2 and 3).

**Table 2:** Presents the chemical composition of the applied compost used in the experiment

Characteristics	Value
pH (1:5)	7.50
EC dS m^−1^ (1:5)	5.10
Organic matter (g kg^−1^)	380
Total N (g kg^−1^)	12.10
Total P (g kg^−1^)	7.00
Total K (g kg^−1^)	8.60
Fe (mg kg^−1^)	2397
Mn (mg kg^−1^)	241
Zn (mg kg^−1^)	46
Cu (mg kg^−1^)	13

**Table 3:** Average chemical characteristics of spent mushroom substrate used in the experiment

Characteristics	Value	Characteristics	Value	Characteristics	Value
Cellulose %	55	Moisture %	30	(Mg) %	0.28
Hemicellulose %	25	(N) %	2.2	(Na) %	0.01
Lignin %	18.5	(P) %	0.5	(Fe) %	0.005
Protein %	9.75	(K) %	0.8	Vit. B12%	0.005
Fat %	1.5	(Ca) %	0.4	Vit. C %	0.01

The phytostimulant treatments (subplots) used, which included both seaweed extract and spirulina extract, were sourced as follows: Seaweed was obtained from Sinochem Agro Co., Ltd., Shanghai, China. While spirulina algae were sourced from Daco Company, Cairo, Egypt. Seaweed extract was prepared by dissolving it in water at the rates (2 and 4 g L^−1^) and spraying it directly on plant leaves. Spirulina algae extract was soaked in water at the rates (2 and 4 g L^−1^) for 3 h before use to ensure complete dissolution, as the authors recommend soaking it for optimal solubility and effect. Phytostimulant treatments were applied as foliar three times: The first was one month after transplanting, and the second and third applications were three weeks apart, respectively, in both seasons. The chemical analysis of both seaweed extract and spirulina algae extract can be seen in (Tables 4 and 5).

**Table 4:** The chemical composition of the used seaweed extract

Characteristics	Value	Characteristics	Value
Organic (N) %	3.06	Mn ppm	11
P_2_O_5_%	2.46	Cu ppm	14
K_2_O %	10	Total amino acid %	5
S %	2.44	Carbohydrates %	29
Ca %	0.23	Alginic acid %	10
Mg %	0.56	IAA %	0.04
Fe ppm	144	Cytokinins %	0.03
Zn ppm	60		

### Determining the chemical characteristics of soil, plant, and compost samples

Prior to transplanting, soil samples were collected from a depth of 0–30 cm using an auger. Samples were air-dried, ground, and sieved through a 2-mm mesh for analysis. Soil texture was determined using the pipette method, as described by [28]. Certain physical and chemical properties of the studied soil were determined. A glass electrode was used to measure the soil pH on a digital pH meter [29]. Soil electrical conductivity (EC) was measured using an EC meter [29]. Soil organic matter content was determined according to the method of [30]. Nitrogen (N) availability was determined following the Kjeldahl method [29]. Soil available phosphorus was determined by extracting with 0.5 N NaHCO_3_ and measuring with a spectrophotometer [31]. Available potassium (K) in the soil was extracted with 1 M ammonium acetate (pH 7.0) and its concentration determined by flame photometry [29]. The total concentrations of nitrogen (N), phosphorus (P), and potassium (K) in digested compost and plant samples were measured after digestion with H_2_O_2_ and H_2_SO_4_ [32].

**Table 5:** The chemical composition of the applied spirulina algae extract

Characteristics	Value	Characteristics	value
Carotenoids and Phytonutrients		Pantothenic acid (mcg)	3
Chlorophyll (mcg)	28	Minerals	
Total carotenoids (mcg)	19	Calcium (mcg)	12
Phytocyanin (mcg)	289	Phosphorus (mcg)	30
Zeaxanthin (mcg)	4	Iodine (mcg)	22
Gamma linolenic (mcg)	33	Magnesium (mcg)	26
Superoxide dismutase (IU)	1040	Zinc (mcg)	0.1
Vitamins		Selenium (mcg)	3
Vitamin A (as beta carotene) (mcg)	3763	Copper (mcg)	0.02
Vitamin k1&k2 (mcg)	51	Manganese (mcg)	0.5
Thiamin (B1) (mcg)	2	Molybdenum (mcg)	< 12
Riboflavin (B2) (mcg)	5	Potassium (mcg)	45
Niacin (B3) (mcg)	237	Iron (mcg)	3
Vitamin B6 (mcg)	21	Protein (%)	60
Folate (mcg)	0.2		
Vitamin 12 (mcg)	8		
Biotin (mcg)	< 1		

### Experimental measurements

#### Vegetative growth characteristics

Twenty-one weeks after planting, the plants were harvested, and their final morphophysiological features are shown in the results. Immediately prior to the final harvest, plant height (cm), number of branches per plant, fresh herb weight (g/plant), and dry herb weight (g/plant) were measured.

####  Flowering characteristics

The number of inflorescences/plants, number of flowers/inflorescences, inflorescence fresh weight, and inflorescence dry weight were recorded at the end of the growing season from five randomly selected plants.

#### Yield component characteristics

To prevent seed loss after flowering, paper cones were carefully constructed and securely attached to each selected plant in late February. This method was used because mature seeds tend to fall off rapidly once they reach full maturity. After the cones were installed, the following crop traits were recorded: seed weight per plant (g/plant) and seed yield per hectare (kg ha^−1^).

#### Chemical constituents

The chemical composition of borage seeds was determined following the official methods of the Association of Official Analytical Chemists [33]. All analyses were performed in triplicate, and the mean values will be reported.).

#### Determination of total Ash content (TAC)

TAC was determined by ashing 1.0–2.0 g samples in a muffle furnace at 600 ± 10 °C for 3 h [33].

#### Determination of fixed oil

The determination of fixed oil or crude lipids followed the method [33]: Precisely weighed dried seed samples (1–2 g) were extracted in a Soxhlet apparatus with petroleum ether (60–80 °C) for 15 h. The petroleum ether was then evaporated under reduced pressure, and the total lipids content was subsequently weighted and recorded.

#### Determination of total nitrogen (TN) and crude protein (CP)

The Kjeldahl procedure [33] was used to determine total nitrogen in the seeds, with analysis performed on a Rapid Nitrogen Apparatus Model-005 (RNAM-005). Crude protein was estimated by multiplying the nitrogen content by a factor of 6.25, using 5.75 specifically for borage seeds.

#### Extraction and determination of total phenolic compounds (TPCs)

The concentration of Total Phenolic Compounds (TPCs) in the methanolic extracts was determined using a modified method based on [34]. A volume of 1 mL of the extract was mixed with 1 ml of Folin-Ciocalteu phenol reagent. After a 3-minute incubation period, 1 ml of a saturated sodium carbonate solution (35%) was added to the mixture, and the volume was adjusted to 10 ml with the addition of distilled water. This reaction mixture was then incubated in the dark for 90 min, and its absorbance was subsequently measured at 725 nm using a spectrophotometer. A calibration curve was constructed using various concentrations of gallic acid (0.01–1 mM) as a standard.

#### Extraction and determination of total flavonoids (TFs)

Oven-dried sample seeds (30 g) were extracted in a Soxhlet extractor with 100 ml distilled ethanol for 1 h and the extract filtered. A known volume of the extract was transferred into a 10 mL volumetric flask. Distilled water was added to bring the volume to 5 mL, followed by the addition of 0.3 mL of NaNO₂ (1:20). After 5 min, 3 mL of AlCl₃ (1:10) was added. Six minutes later, 2 mL of 1 mol L⁻¹ NaOH was added, and the final volume was made up to 10 mL with distilled water. The mixture was thoroughly shaken, and the absorbance was measured at 510 nm against a reagent blank using a spectrophotometer (Taizhou Radio Factory) and the total flavonoid content was determined as Catechin equivalent (CE) (6.25–200.00 µg/mL) and was expressed as g of CE/100 g according to the method described by [34].

#### Statistical analyses

The significance of differences between the treatments was statistically assessed using Analysis of Variance (ANOVA) [two-way]. Differences between individual means were separated by Duncan’s multiple range test at a probability level of 0.05. All statistical analyses of the results were performed using the Costat software [35]. Principal component analysis (PCA) utilizing Origin Lab (2019b) was conducted to evaluate the impacts of compost, spent mushroom substrate, and foliar phytostimulant extracts on the interrelations among key soil variables, plant vegetation, flowering, yield, nutrients, and chemical constituents of the borage plant.

## Results

### Soil chemical properties

The impacts of compost and spent mushroom substrate (SMS) on some soil chemical properties in 2021 and 2022 are shown in (Table 6). In both years, compost and mushrooms played a significant role in influencing the soils chemical properties. Compost and mushroom applied slightly decreased the soil pH, and significantly increased soil salinity (EC), and soil organic matter (SOM), in both years in comparison with the control treatment. Adding mushroom decreases pH by 1.48 and 1.60% also, it increases the SOM by 84.65 and 87.48%, while compost increased EC by 32.88 and 33.33% in the two experimental seasons.

Foliar application of phytostimulant (seaweed and spirulina algae) extracts affected soil pH by contributing to its decline. However, EC and SOM did not show significant changes during the first and second growing seasons when different phytostimulants were applied, compared to the control treatment. Also, in the two growing seasons, the interaction among mushroom, compost, and foliar spray applications by phytostimulant extract significantly affected soil pH, EC, and SOM. The greatest reduction in soil pH was recorded with the combination of mushroom waste and spirulina at 4 g L^−1^. Additionally, SOM increased by 85.95% and 89.42% in the first and second growing seasons, respectively, under the mushroom waste and spirulina (4 g L^−1^) treatment compared to the control.

**Table 6:** Effect of organic matters and phytostimulants on some soil chemical properties after cultivated borage plants

Treatment			First season			Second season		
			Ph	EC	OM (g kg^−1^)	pH	EC	OM (g kg^−1^)
Organic matter A		C	8.13	0.72 C	6.58 C	8.13	0.73 C	6.55 C
		Compost	8.08	0.96 A	9.78B	8.06	0.97 A	9.92B
		Mushroom	8.01	0.86B	12.15 A	8.00	0.87B	12.28 A
Phytostimulant extracts B		C	8.08	0.85 A	9.43 A	8.07	0.86 A	9.51 A
		Seaweed 2 g L^−1^	8.08	0.84 A	9.50 A	8.07	0.85 A	9.59 A
		Seaweed 4 g L^−1^	8.07	0.84 A	9.52 A	8.06	0.86 A	9.59 A
		Spirulina 2 g L^−1^	8.07	0.85 A	9.54 A	8.06	0.87 A	9.62 A
		Spirulina 4 g L^−1^	8.07	0.85 A	9.57 A	8.06	0.87 A	9.66 A
Interaction	Control	C	8.14	0.73c	6.55c	8.14	0.74c	6.52c
		Seaweed 2 g L^−1^	8.13	0.70c	6.58c	8.13	0.71c	6.56c
		Seaweed 4 g L^−1^	8.13	0.69c	6.58c	8.13	0.72c	6.55c
		Spirulina 2 g L^−1^	8.13	0.73c	6.59c	8.13	0.75c	6.56c
		Spirulina 4 g L^−1^	8.12	0.73c	6.58c	8.12	0.74c	6.55c
	Compost	C	8.09	0.96a	9.61b	8.07	0.97a	9.74b
		Seaweed 2 g L^−1^	8.08	0.95a	9.78b	8.06	0.97a	9.92b
		Seaweed 4 g L^−1^	8.08	0.96a	9.84b	8.06	0.97a	9.98b
		Spirulina 2 g L^−1^	8.08	0.97a	9.88b	8.06	0.98a	10.02b
		Spirulina 4 g L^−1^	8.07	0.97a	9.94b	8.05	0.99a	10.07b
	SMS	C	8.02	0.86b	12.12a	8.01	0.87b	12.26a
		Seaweed 2 g L^−1^	8.01	0.86b	12.14a	8.00	0.87b	12.28a
		Seaweed 4 g L^−1^	8.01	0.86b	12.14a	8.00	0.88b	12.25a
		Spirulina 2 g L^−1^	8.01	0.85b	12.17a	8.00	0.87b	12.28a
		Spirulina 4 g L^−1^	8.00	0.86b	12.18a	7.99	0.88b	12.35a
		LSD	A = 0.004	A = 0.04	A = 0.27	A = 0.004	A = 0.04	A = 0.26
			B = 0.009	B = 0.03	B = 0.22	B = 0.010	B = 0.03	B = 0.20
			A×B = 0.017	A×B = 0.06	A×B = 0.38	A×B = 0.017	A×B = 0.06	A×B = 0.35

Means in each column followed by the same letters are not significantly different (P < 0.05) by Duncan’s multiple range test

*SMS* Spent mushroom substrate, *C* control, *pH* soil reaction, *EC* electrical conductivity, *OM* organic matter. All values are the mean of three replicates

### Soil nutrient availability

**Fig. 2:**
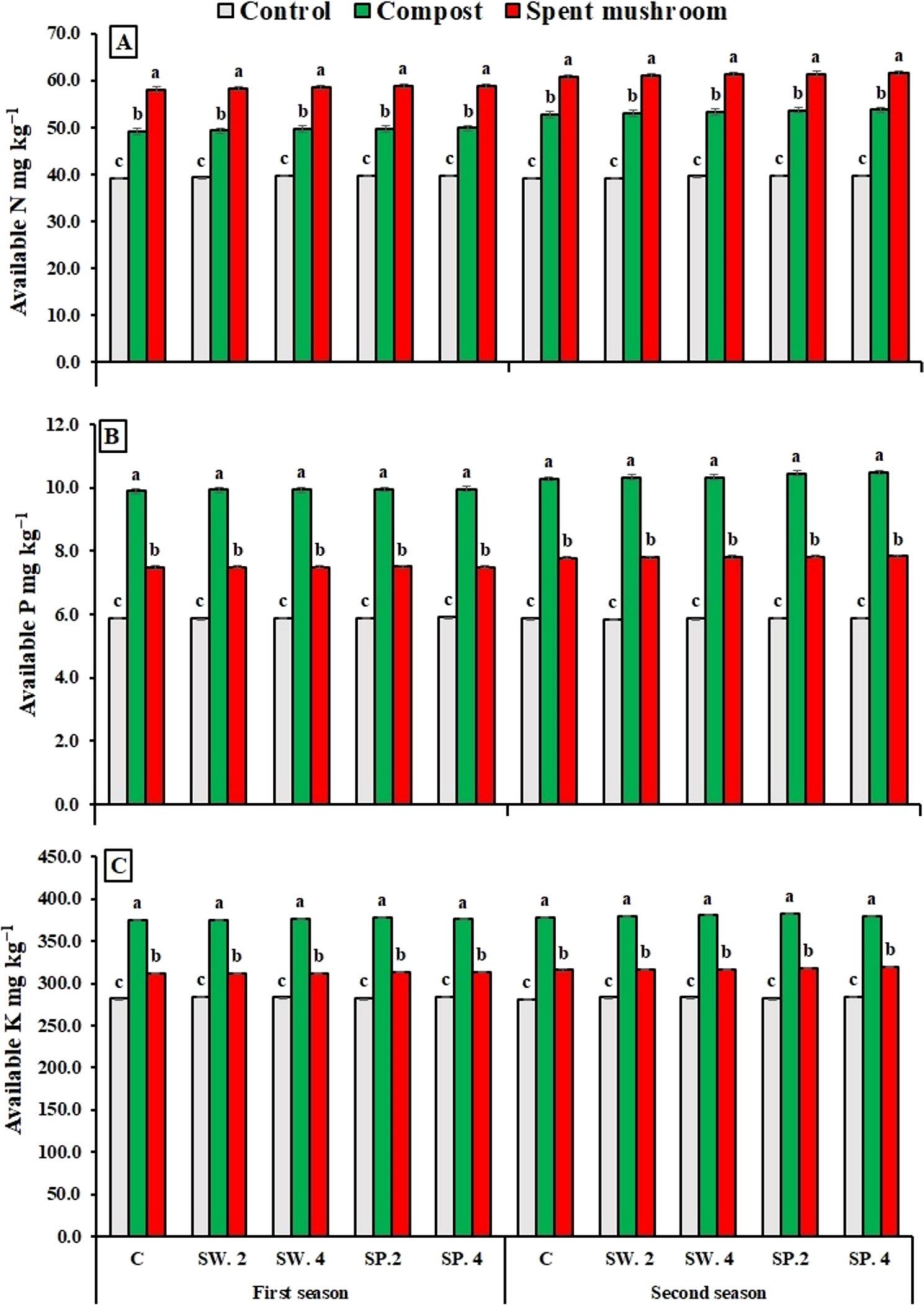
Effect of organic matters and phytostimulants on nutrient availability of nitrogen N in (**A**), (**B**) phosphorus P and (**C**) potassium K of borage plants on the average basis of the two seasons. C = control, SW (2 and 4) = seaweed extract g/L, and SP (2 and 4) = spirulina g/L. Means in each column followed by the same letters are significantly different (*P* < 0.05) by Duncan’s multiple range test

Data presented in Fig. 2 indicates that the application of organic fertilizers, specifically compost and spent mushroom substrate, significantly enhanced nutrient availability across both growing seasons. The use of spent mushroom substrate led to notable increases in available nitrogen (N), by 48.03% and 54.93% in the first and second seasons, respectively. Compost application resulted in substantial improvements in available phosphorus (P), with increases of 68.71% and 76.32%, and in available potassium (K), with increases of 32.88% and 34.34%, compared to the control treatment in the first and second seasons, respectively.

The results indicated that foliar application of phytostimulant extracts, particularly those with a high concentration of spirulina, led to a notable enhancement in nutrient availability (Fig. 2). Specifically, N increased by 1.50–1.57%, P by 0.43–1.06%, and K by 0.64–0.85% compared to the control treatment across both the first and second growing seasons.

The combined application of compost and high-dose spirulina significantly enhanced nutrient availability (Fig. 2). P levels increased by 69.55% and 78.53%, while K levels rose by 33.37% and 35.73% in the first and second seasons, respectively, compared to the control. The most substantial increase in N was recorded with the application of mushroom waste combined with spirulina at 4 g L^−1^, resulting in a 50.44% and 57.42% increase over the control in the first and second seasons, respectively.

### Vegetative characteristics

Data presented in (Tables 7 and 8) reveal that plant height, number of branches per plant, fresh herb weight (g/plant), and dry herb weight (g/plant) of borage were significantly enhanced by the application of various organic fertilizers across both experimental seasons. The use of organic amendments resulted in a marked improvement in all measured growth parameters compared to the control treatment. Among the tested fertilizers, compost and spent mushroom substrate demonstrated the most pronounced effects. Specifically, the spent mushroom substrate increased plant height by 20.42–21.25%, fresh herb weight by 24.78–31.17%, and dry herb weight by 25.05–31.18% over the control in the first and second seasons, respectively.

Tables 7 and 8 indicate that foliar application of phytostimulant extracts significantly enhanced plant height, number of branches per plant, fresh herb weight, and dry herb weight of borage during both growing seasons. All tested materials and concentrations led to statistically significant improvements (*p* < 0.05) in these growth parameters compared to the control, confirming the positive impact of phytostimulant treatments on vegetative development. The most pronounced improvements in borage growth were observed with foliar application of spirulina extract at the highest concentration (4 g L^−1^). This treatment significantly increased plant height by 21.9% and 20.4%, branch number by 33.3% and 33.9%, fresh herb weight by 30.9% and 40.0%, and dry herb weight by 30.9% and 40.0% in the first and second seasons, respectively, compared to the control.

The interaction between the two study factors had a statistically significant effect on all measured borage growth traits, except for branch number, across both seasons (Tables 7 and 8). Treatments combining spent mushroom substrate with foliar application of spirulina at 4 and 2 g L^−1^ consistently produced the highest improvements in most parameters.

**Table 7:** Effect of organic matters and phytostimulants on plant height, branch number and herb fresh weigh (g/plant) of borage plants during the two growing seasons

Treatments			First season			Second season		
			Plant height (cm)	Branch number	Herb Fresh weight (gplant^−1^)	Plant height (cm)	Branch number	Herb fresh weight (g plant^−1^)
Organic matter A		C	75.9 C	10.8B	754.8 C	79.5 C	11.8B	781.3 C
		Compost	87.5B	12.3 A	851.3B	91.7B	13.4 A	872.1B
		SMS	91.4 A	12.5 A	941.9 A	96.4 A	13.6 A	1024.9 A
Phytostimulant B		C	76.8D	9.9D	742.3E	80.8E	10.9 C	748.2D
		Seaweed 2 g L^−1^	81.5 C	11.5 C	789.4D	86.3D	12.7B	839.6 C
		Seaweed 4 g L^−1^	85.8B	12.1BC	861.0 C	89.1 C	12.9B	902.9B
		Spirulina 2 g L^−1^	86.9B	12.6AB	882.1B	92.8B	13.6AB	925.4B
		Spirulina 4 g L^−1^	93.6 A	13.2 A	971.9 A	97.3 A	14.6 A	1047.6 A
Interaction	Control	C	69.9k	8.8e	646.4f	74.7 h	10.5de	655.0 g
		Seaweed 2 g L^−1^	72.2jk	9.9de	697.7e	76.1gh	10.9de	725.7f
		Seaweed 4 g L^−1^	77.7i	11.2 cd	785.4d	81.0f	11.7cde	808.6e
		Spirulina 2 g L^−1^	78.8hi	11.7bc	798.7d	81.2ef	12.4bcd	829.0de
		Spirulina 4 g L^−1^	80.9ghi	12.4bc	845.9c	84.7e	13.3bc	888.1 cd
	Compost	C	76.9ij	9.9de	782.8d	79.3 fg	10.3e	788.5e
		Seaweed 2 g L^−1^	84.0efg	12.5abc	821.6 cd	89.7 cd	13.5abc	843.3de
		Seaweed 4 g L^−1^	90.7 cd	12.8ab	825.7 cd	91.8bcd	13.7abc	836.0de
		Spirulina 2 g L^−1^	87.5def	13.0ab	846.1c	94.7b	14.3ab	879.3d
		Spirulina 4 g L^−1^	98.3ab	13.2ab	980.6b	103.1a	15.3a	1013.3b
	SMS	C	83.5fgh	11.0 cd	797.7d	88.3d	11.8cde	801.3e
		Seaweed 2 g L^−1^	88.3de	12.1bc	848.8c	93.1bc	13.7abc	949.7c
		Seaweed 4 g L^−1^	89.0d	12.4abc	972.0b	94.4b	13.3bc	1064.1b
		Spirulina 2 g L^−1^	94.4bc	13.1ab	1001.5b	102.4a	14.0ab	1068.0b
		Spirulina 4 g L^−1^	101.7	13.9	1089.3	104.0	15.3	1241.3
		LSD	A = 2.9	A = 0.5	A = 36.7	A = 1.7	A = 0.9	A = 30.6
			B = 2.7	B = 0.9	B = 24.8	B = 2.1	B = 1.2	B = 35.8
			*A×B = 4.8*	*A×B = N.S*	*A×B = 42.9*	*A×B = 3.6*	*A×B = N.S*	*A×B = 61.9*

All values are the mean of three replicates. Means in each column followed by the same letters are not significantly different (P < 0.05) by Duncan’s multiple range test

*SMS* Spent mushroom substrate, *C* control

**Table 8:** Effect of organic matters and phytostimulants on herb dry weight (g/plant), inflorescence number/plant and flower number/inflorescence of borage plants during the two growing seasons

Treatments			First season			Second season		
			Herb dry weight (g plant^−1^)	Inflorescence number/plant	Flower number/inflorescence	Herb dry weigh (g plant^−1^)	Inflorescence numberplant^−1^	Flower number/inflorescence
Organic matter A		C	130.1 C	13.8 C	12.7B	134.7 C	14.4 C	13.2 C
		Compost	146.8B	16.0B	13.3AB	150.4B	16.8B	13.6B
		SMS	162.7 A	18.8 A	13.8 A	176.7 A	20.2 A	14.2 A
Phytostimulant B		C	128.1D	12.9 C	10.6D	129.0 A	14.6 C	11.7D
		Seaweed 2 g L^−1^	136.2 C	15.6B	13.2 C	144.7 C	16.0BC	13.3 C
		Seaweed 4 g L^−1^	148.6B	16.8AB	13.6BC	155.7B	17.7B	13.8BC
		Spirulina 2 g L^−1^	152.2B	17.1AB	14.3AB	159.6B	17.4B	14.5AB
		Spirulina 4 g L^−1^	167.7 A	18.6 A	14.6 A	180.6 A	20.1 A	15.0 A
Interaction	Control	C	111.5f	11.3f	9.6f	112.9 g	12.9d	11.2 g
		Seaweed 2 g L^−1^	120.3e	12.2ef	12.7 cd	125.1f	12.7d	13.0def
		Seaweed 4 g L^−1^	135.4d	15.1 cd	13.2bcd	139.4e	16.0 cd	13.3de
		Spirulina 2 g L^−1^	137.7d	14.8cde	13.8abc	142.9de	13.8 cd	14.1bcd
		Spirulina 4 g L^−1^	145.9c	15.4 cd	14.2abc	153.1 cd	16.7bc	14.5abcd
	Compost	C	135.0d	12.9def	10.9ef	135.9e	14.4 cd	11.6 fg
		Seaweed 2 g L^−1^	141.6 cd	15.8c	13.3bcd	145.4de	15.9 cd	13.4cde
		Seaweed 4 g L^−1^	142.4 cd	15.9c	13.6abc	144.1de	16.3bc	13.7bcde
		Spirulina 2 g L^−1^	145.9c	16.3bc	14.2abc	151.6d	16.8bc	14.4abcd
		Spirulina 4 g L^−1^	169.1b	18.9ab	14.4abc	174.7b	20.7a	14.9abc
	SMS	C	137.8d	14.5cde	11.5de	138.1e	16.6bc	12.2efg
		Seaweed 2 g L^−1^	146.6c	18.7ab	13.7abc	163.7c	19.5ab	13.6bcde
		Seaweed 4 g L^−1^	167.9b	19.4a	14.0abc	183.5b	20.7a	14.2abcd
		Spirulina 2 g L^−1^	173.0b	20.2a	14.8ab	184.1b	21.5a	15.1ab
		Spirulina 4 g L^−1^	188.2a	21.4a	15.3a	214.0a	22.8a	15.7a
		LSD	A = 6.0	A = 1.0	A = 0.8	A = 5.3	A = 1.7	A = 0.3
			B = 4.3	B = 1.4	B = 1.0	B = 6.2	B = 2.0	B = 0.9
			A×B = 7.5	A×B = N.S	A×B = N.S	A×B = 10.7	A×B = N.S	A×B = N.S

All values are the mean of three replicates. Means in each column followed by the same letters are not significantly different (*P* < 0.05) by Duncan’s multiple range test

*SMS* Spent mushroom substrate, *C* control

### Flowering traits

**Fig. 3:**
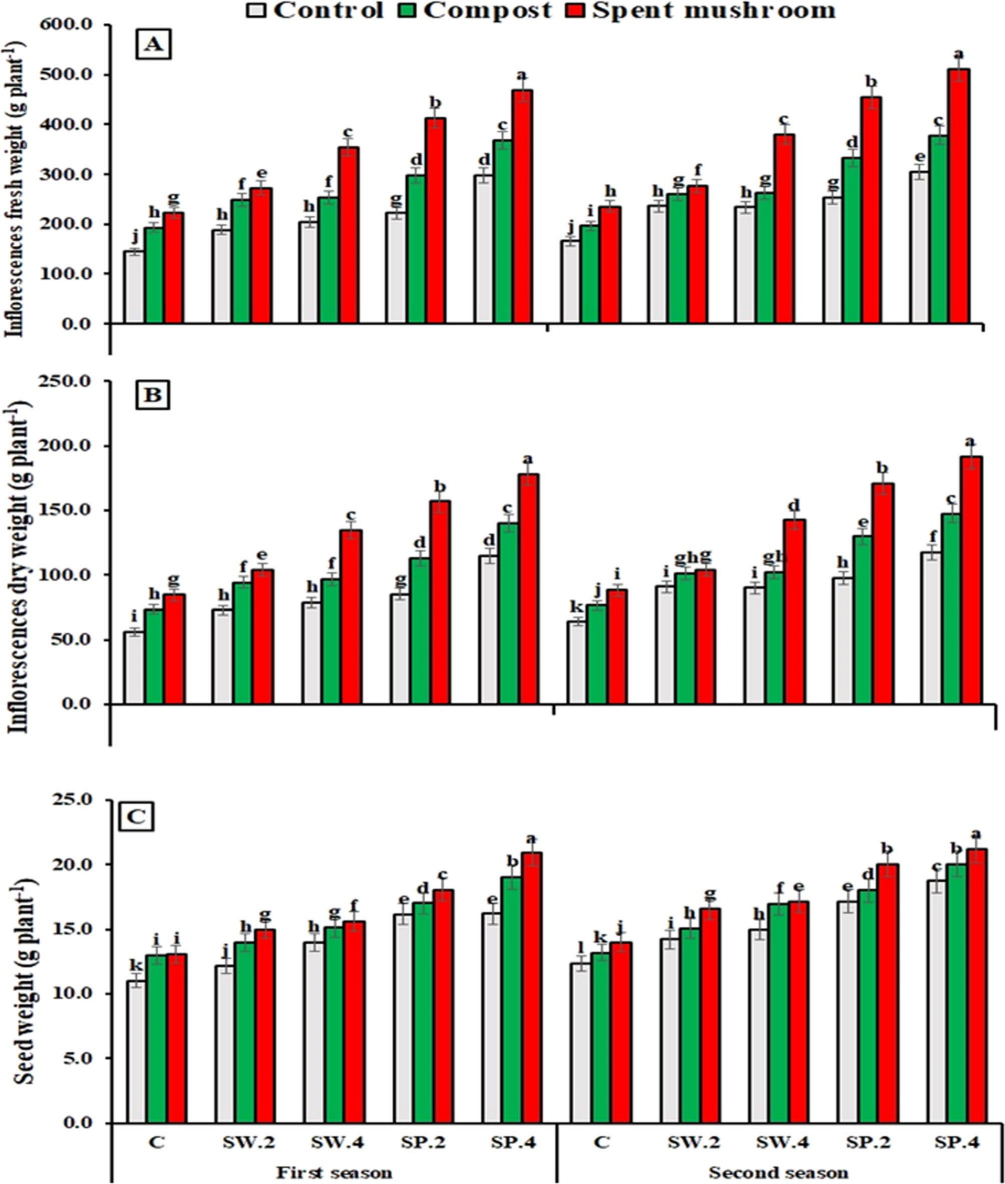
Effect of organic matters and phytostimulants on inflorescences fresh weight (**A**), (**B**) inflorescences dry weight, and (**C**) seed weight of borage plants on the average basis of the two seasons. C = control, SW (2 and 4) = seaweed extract g/L, and SP (2 and 4) = spirulina g/L. Means in each column followed by the same letters are significantly different (*P* < 0.05) by Duncan’s multiple range test

Data presented in (Fig. 3) shows that different organic amendments significantly influenced flowering traits of borage in both seasons, including the number of inflorescences/plants, number of flowers/inflorescences, and inflorescence fresh and dry weights. Compost and spent mushroom waste notably improved these parameters, except for flower number/inflorescence in the first season. Among treatments, spent mushroom waste consistently produced the best results. Applied at 6.4 t ha⁻¹, it increased the number of inflorescences by 36.2% and 40.3%, flower number by 8.7% and 7.6%, fresh weight by 64.0% and 55.6%, and dry weight by 61.9% and 51.7% over the control in the first and second seasons, respectively.

Observations across both seasons showed that phytostimulant treatments significantly enhanced flowering traits in borage, including the number of inflorescences/plants, flowers/inflorescence, and inflorescence fresh and dry weights, except for seaweed extract at 2 g L^−1^, which had no significant effect on inflorescence number in the second season (Fig.3). Spirulina extract at 4 g L^−1^ consistently produced the best results, increasing inflorescence number by 44.2% and 37.7%, flower number by 37.7% and 28.2%, fresh weight by 107.1% and 99.9%, and dry weight by 102.2% and 99.7% over the control in the first and second seasons, respectively.

The interaction between the two studied factors had no significant effect on the number of inflorescences/plant or flowers/inflorescence in either season. However, it significantly influenced inflorescence fresh and dry weights. The highest flowering performance was achieved by combining spent mushroom waste (6.4 t ha⁻¹) with foliar spraying of spirulina extract at 4 g L^−1^, outperforming all other treatments in both seasons (Fig. 3).

### Seed yield

As shown in (Fig. 3) organic amendments significantly boosted borage seed yield in both seasons compared to the untreated control. The highest yield was achieved with spent mushroom waste at 6.4 t ha⁻¹, resulting in increases of 18.85% and 14.99% in the first and second seasons, respectively.

Foliar application of seaweed and spirulina extracts significantly increased borage seed yield in both seasons (Fig. 3). All concentrations tested surpassed the control, with 4 g L^−1^ spirulina achieving the highest yield increase, at 51.46% and 51.78% in the first and second seasons, respectively. The combined treatments significantly improved borage seed yield across both seasons. Notably, the combination of spent mushroom waste (6.4 t ha⁻¹) with spirulina extract (4 g L^−1^) consistently produced the highest seed yield, outperforming all other interaction treatments and the control (Fig. 3).

### Organic and foliar effects on borage NPK nutrient

**Fig. 4:**
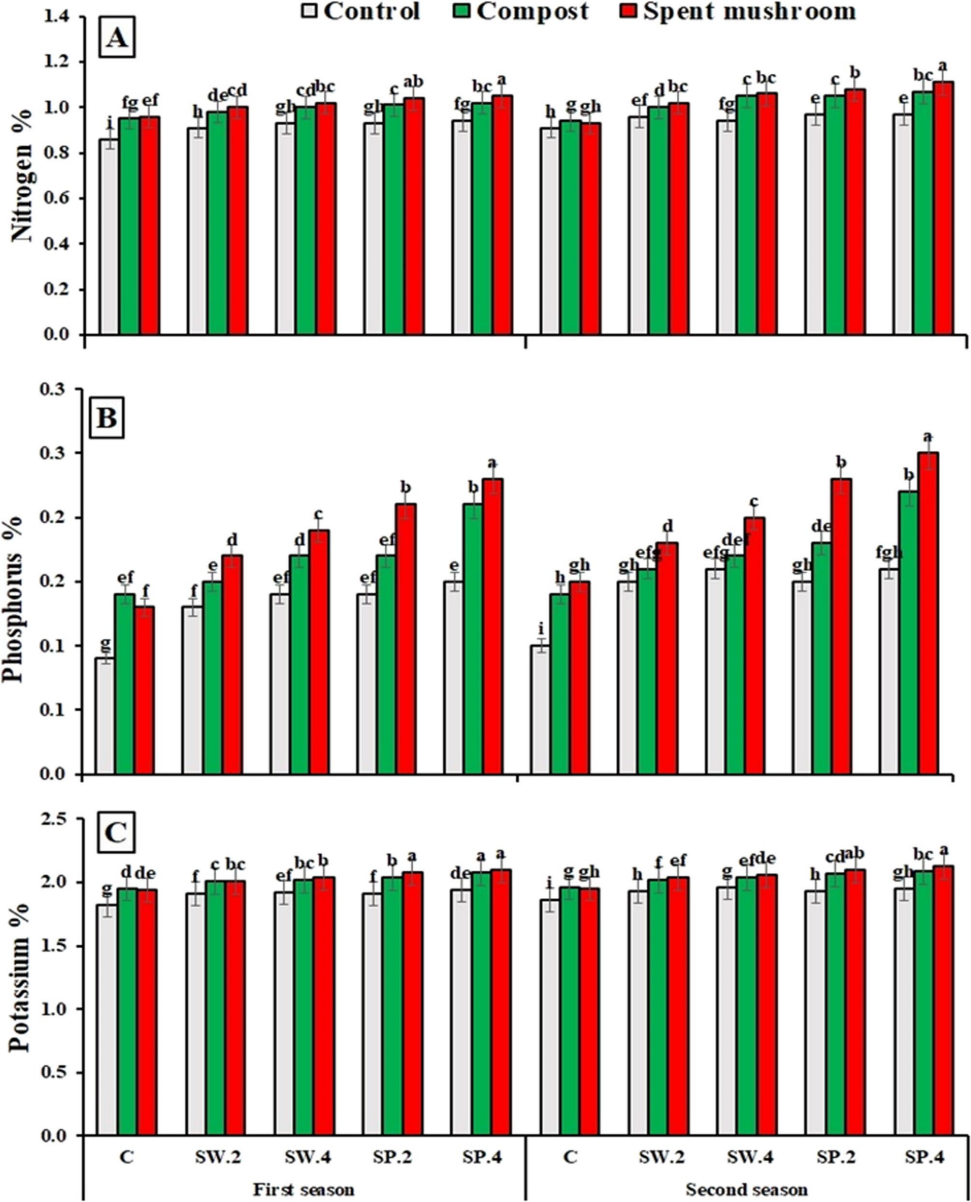
Effect of organic matters and phytostimulants on total nutrient content of nitrogen in (**A**), (**B**) phosphorus and (**C**) potassium of borage plants on the average basis of the two seasons. C = control, SW (2 and 4) = seaweed extract g/L, and SP (2 and 4) = spirulina g/L. Means in each column followed by the same letters are significantly different (*P* < 0.05) by Duncan’s multiple range test

Data presented in (Fig. 4) illustrates that the application of compost and spent mushroom waste significantly increased leaf concentrations of (NPK) in borage plants. compared to the unfertilized control. The highest nutrient levels were observed in plants treated with SMS at 6.4 t ha⁻¹, with increases of N: 10.99% and 9.47%; P: 46.15% and 42.86%; and K: 6.84% and 6.77% in the first and second seasons, respectively. These results highlight the efficacy of SMS as a sustainable organic amendment for enhancing macronutrient uptake in borage cultivation.

Data from (Fig. 4) indicate that foliar application of phytostimulants significantly enhanced N, P, and K concentrations in borage plants. leaves across both seasons, compared to the untreated control. Among the tested treatments, Spirulina algae extract at 4 g/L produced the highest nutrient levels, with increases of N: 8.70% and 12.90%; P: 66.67% and 61.54%; and K: 7.37% and 6.77% in the first and second seasons, respectively.

The integrated application of organic and phytostimulants exhibited a synergistic effect on leaf nutrient accumulation in borage as shown in Fig. 4. Across both seasons, the combination of spent mushroom waste (6.4 t ha⁻¹) with Spirulina algae extract (4 g L^−1^) consistently resulted in the highest concentrations of N, P, and K, outperforming all other treatment combinations. Notably, this enhancement was statistically significant for all nutrients except nitrogen in the first season, where the increase did not reach significance.

### Chemical properties of borage seeds

**Fig. 5:**
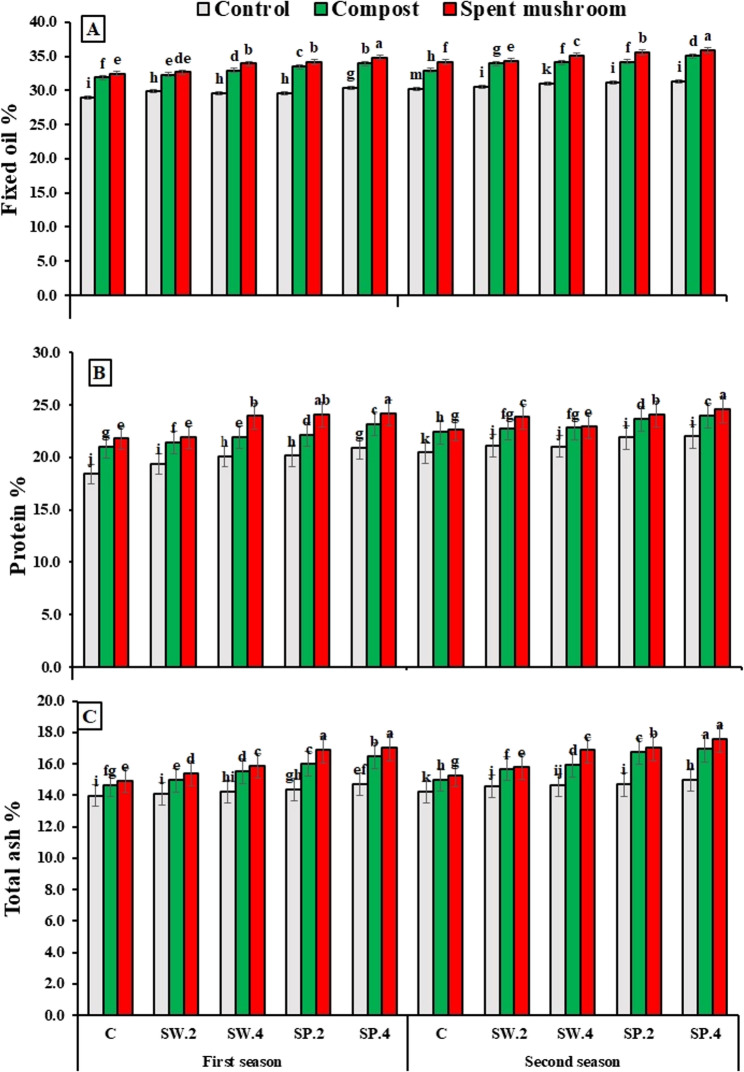
Effect of organic matters and phytostimulants on fixed oil in (**A**), (**B**) protein, and (**C**) total ash of borage plants on the average basis of the two seasons. C = control, SW (2 and 4) = seaweed extract g/L, and SP (2 and 4) = spirulina g/L. Means in each column followed by the same letters are significantly different (*P* < 0.05) by Duncan’s multiple range test

Data presented in (Fig. 5; Table 9) show that both organic amendments (compost and spent mushroom substrate) and phytostimulants (seaweed and spirulina extracts) significantly improved the biochemical composition of borage seeds across two growing seasons. All treatments enhanced fixed oil, protein, total ash, total phenolic compounds (TPCs), and total flavonoids (TFs) compared to the control. Among organic amendments, spent mushroom substrate at 6.4 t ha⁻¹ produced the greatest increases: fixed oil by 13.16% and 13.62%, protein by 17.24% and 10.94%, total ash by 12.25% and 12.85%, TPCs by 10.87% and 16.31%, and TFs by 25.0% and 22.2% in the first and second seasons, respectively. Similarly, among phytostimulants, spirulina extract outperformed seaweed extract, raising fixed oil by 6.23% and 5.02%, protein by 11.57% and 7.60%, total ash by 10.81% and 11.40%, TPCs by 18.32% and 12.68%, and TFs by 50.0% and 29.1% in the respective seasons.

Our results, presented in (Fig. 5; Table 9) illustrate that the interaction between organic amendments and phytostimulants significantly influenced fixed oil, protein, total ash, and total phenolic compounds (TPCs) in borage seeds during both seasons, while total flavonoids (TFs) showed no significant interaction effect. Most combined treatments outperformed the control, with the highest values recorded when spent mushroom substrate (6.4 t ha⁻¹) was combined with 4 g L^−1^ spirulina algae extract, consistently surpassing other combinations across both seasons.

**Table 9:** The effect of organic matters and phytostimulants on TPCs and total TFs percentages of borage plants during the two growing seasons

Treatments			First season		Second season	
			TPCs%	TFs%	TPCs%	TFs%
Organic matter A		Control	1.38 C	0.016B	1.41 C	0.018 C
		Compost	1.47B	0.019 A	1.54B	0.021B
		SMS	1.53 A	0.020 A	1.64 A	0.022 A
Phytostimulant B		C	1.31E	0.014 C	1.42D	0.017 C
		Seaweed 2 g L^−1^	1.45D	0.018B	1.48 C	0.020B
		Seaweed 4 g L^−1^	1.49 C	0.018AB	1.57B	0.020B
		Spirulina 2 g L^−1^	1.51B	0.020AB	1.57B	0.021B
		Spirulina 4 g L^−1^	1.55 A	0.021 A	1.60 A	0.022 A
Interaction	Control	C	1.25j	0.012d	1.35i	0.014 g
		Seaweed 2 g L^−1^	1.38gh	0.016 cd	1.42gh	0.018f
		Seaweed 4 g L^−1^	1.41 fg	0.016 cd	1.41 h	0.018f
		Spirulina 2 g L^−1^	1.40 g	0.017bc	1.42gh	0.018f
		Spirulina 4 g L^−1^	1.45f	0.018bc	1.44 fg	0.019ef
	compost	C	1.31i	0.015 cd	1.41 h	0.018f
		Seaweed 2 g L^−1^	1.44f	0.018bc	1.46f	0.020de
		Seaweed 4 g L^−1^	1.50e	0.019abc	1.61c	0.021 cd
		Spirulina 2 g L^−1^	1.54 cd	0.020ab	1.60c	0.022bc
		Spirulina 4 g L^−1^	1.55c	0.021ab	1.63c	0.023ab
	SMS	C	1.36 h	0.016 cd	1.52e	0.019ef
		Seaweed 2 g L^−1^	1.52de	0.019abc	1.57d	0.022bc
		Seaweed 4 g L^−1^	1.55c	0.020ab	1.68b	0.022bc
		Spirulina 2 g L^−1^	1.59b	0.021ab	1.70ab	0.023ab
		Spirulina 4 g L^−1^	1.64a	0.022a	1.72a	0.024a
		LSD	A = 0.01	A = 0.001	A = 0.01	A = 0.002
			B = 0.02	B = 0.001	B = 0.02	B = 0.001
			A×B = 0.03	A×B = N.S	A×B = 0.03	A×B = N.S

Means in each column followed by the same letters are not significantly different (*P* < 0.05) by Duncan’s multiple range test

All values are the mean of three replicates

*SMS* Spent mushroom substrate, *C* control, *TPCs*% Total phenolic compounds, *TFs*% Total flavonoids

### Principal component analysis

**Fig. 6:**
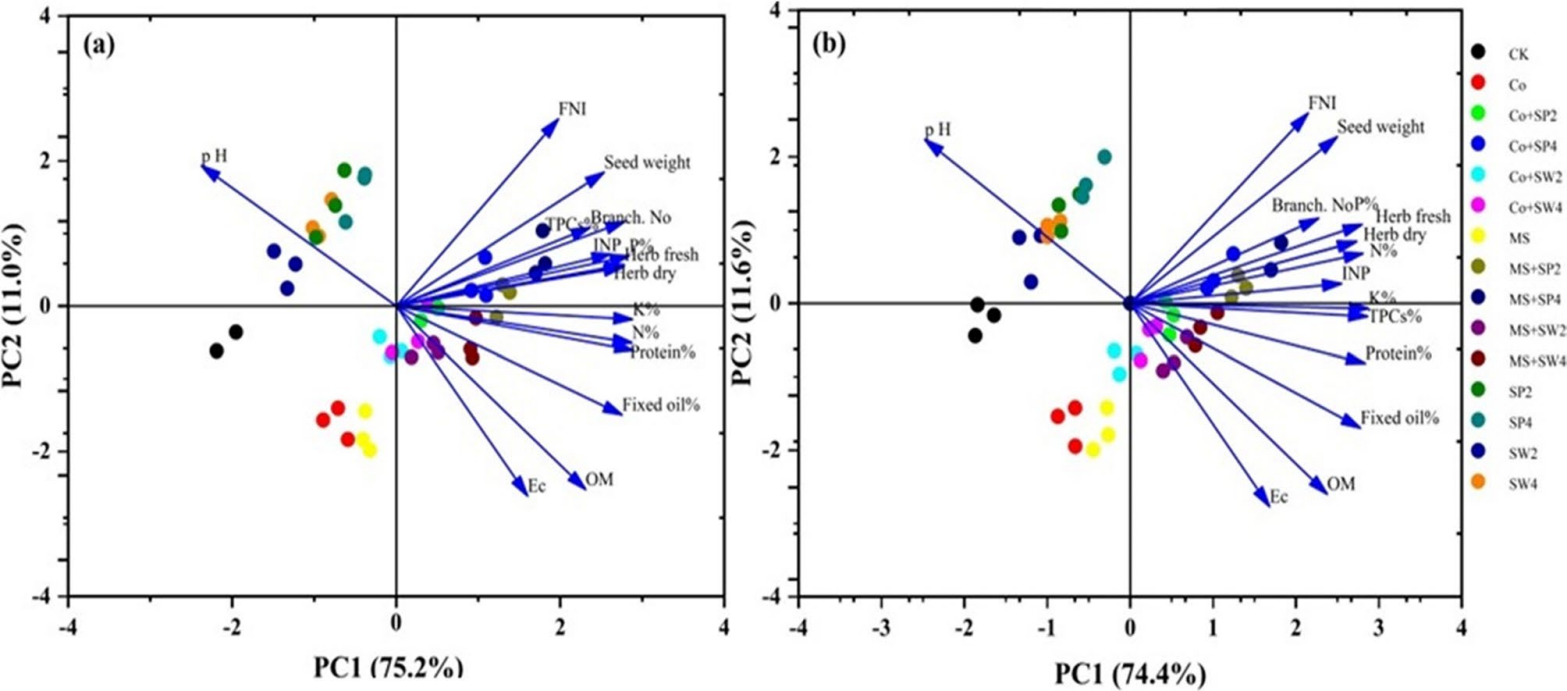
Multivariate principal component analysis (PCA) illustrating the effects of compost, spent mushroom substrate, alone or combined with foliar phytostimulant extracts on organic matter (OM), soil pH, electrical conductivity (EC), branch number, herb fresh weight, herb dry weight, inflorescence number plant (INP), flower number inflorescence (FNI), seed weight, nutrient concentrations (N, P, and K percentages in borage plants), fixed oil percentage, protein percentage, and total phenolic compounds (TPCs). (**a**) represents data from the first season, and (**b**) represents data from the second season

Principal component analysis (PCA) was employed to evaluate the impact of individual and combined applications of compost, spent mushroom substrate, and foliar phytostimulant extracts on key soil properties, vegetation characteristics, flowering, yield, nutrient concentrations, and active constituents of borage plants (Fig. 6a and b). Across both seasons, the initial two principal components demonstrate considerable significance, explaining 86.2% and 86% of the total variance, respectively. The PCA revealed distinct clusters linking specific treatments to key traits. Combined applications of spent mushroom substrate or compost with 4 g L⁻¹ spirulina algae extract and spent mushroom substrate with 2 g L⁻¹ spirulina were strongly associated with higher TPCs, yield components (branch number, herbage biomass, seed weight), phosphorus content, and flowering performance, primarily driven by PC1 (Fig. 6a and b). Conversely, treatments combining compost or spent mushroom substrate with seaweed extract (2–4 g L⁻¹) and compost with 2 g L⁻¹ spirulina correlated with improved soil properties (organic matter, EC), nutrient availability (N, K), and active constituents (protein, fixed oil), reflecting PC1 positive and PC2 negative loadings. PC2 was largely associated with soil pH, which clustered with individual applications of spirulina, seaweed extracts, and the control (Fig. 6). Overall, PCA underscores the synergistic role of organic matter and phytostimulants in enhancing soil fertility, plant performance, and bioactive compound accumulation in borage.

## Discussion

### Effect of experiment treatments on soil properties

Our results showed that compost and spent mushroom waste addition, either individually or combined with phytostimulant extracts, affected some soil chemical properties. The reduction in soil pH following mushroom waste application in alkaline, semi-arid soils can be attributed to rhizosphere acidification. This process occurs as plant roots absorb more cations than anions, releasing protons (H⁺) into the rhizosphere, which lowers soil pH [36]. Such acidification enhances nutrient availability and may improve soil conditions for borage growth [37, 38]. Moreover, organic matter can buffer changes in soil pH by releasing hydrogen ions (H^+^) during decomposition, leading to a reduction in pH. The production of organic acids through the mineralization of organic materials could decrease soil pH [39]. Also, the application of mushrooms decreases pH in silty loam and sandy soil textures [40].

Compost-amended soil had higher EC than mushroom waste-treated soil, likely due to compost’s greater soluble ion content. Furthermore, the observed increase in salinity of the soil could be a sign of salt buildup and the replenishment of Na + adsorbed from soil particles and functional groups such as hydroxyl and carboxylate [41, 42]. Compost added to soil increased soil salinity by 10.90% in clay loam soil [13].

Organic matter enhances soil chemical properties, water retention, nutrient availability, and improves soil pH [43]. Mushroom waste is particularly effective due to its high nutrient content and the presence of beneficial microorganisms that enhance nutrient availability by plants [44–46]. The addition of wasted mushrooms raised the soil’s organic carbon content [47]. This explains why mushroom waste was more effective than compost in increasing some nutrient availability. Besides, it also contains macronutrients such as nitrogen, phosphorus, and potassium, as well as other micronutrients [48, 49].

Furthermore, the application of the mushroom elevated the nitrogen concentration, this fact could be due to the biological fixation of nitrogen in the soil, which may be associated with immobilization processes and the proliferation of free-living N_2_-fixing bacteria as a result of utilizing mushroom waste [11, 47, 50]. Additionally, the slow release of nitrogen from the decomposing mushrooms supports efficient nitrogen uptake by plants [51, 52]. These results were correlated with the refer of [53] who showed that the addition of 100 t ha^−1^ spent mushroom substrate increased yield, nitrogen, and phosphorus content by 50%, 28%, and 230% respectively, while potassium is increased by three times in the soil after harvest. The amount of P that was accessible in the soil was raised by applying spent mushroom substrate by 1.3–1.6 times [47]. Algae extracts can enhance nutrient content and assimilation, leading to higher concentrations of essential nutrients in plant tissues [54]. This is consistent with findings from other studies that highlight the role of compost and spent mushroom waste in improved soil fertility, which led to enhanced plant growth [16, 55].

### Effect of experiment treatments on growth and biochemical traits

Organic materials are particularly beneficial as they supply readily available nutrients, enhance phytohormone synthesis, improve mineral solubility, and promote nitrogen uptake, collectively stimulating plant growth and development [56]. Spent mushroom substrate (SMS) is a valuable organic material that improves soil structure, water retention, nutrient availability, and microbial activity, thereby supporting root development and crop performance [57]. Compost application has been shown to enhance growth, yield, oil content, and oil composition in medicinal plants such as Nigella sativa, likely through activation of metabolic processes and enzyme system [58–60]. Similarly, SMS contributes essential nutrients (N, P, K) and humus, improving soil fertility and plant response to fertilization [61, 62]. Studies indicate that mushroom straw waste increases protein content, modifies oil composition, and extends flowering duration, resulting in higher yields and improved nutritional quality of medicinal plants [63]. These properties highlight SMS as a sustainable organic fertilizer for enhancing crop productivity and quality.

This study demonstrates that phytostimulants significantly improved borage growth, yield, and biochemical composition, with spirulina extract consistently outperforming seaweed extract across both seasons. The superior effect of spirulina is attributed to its rich composition of bioactive compounds, including essential amino acids, vitamins, minerals, polyunsaturated fatty acids, pigments, and antioxidants, which enhance metabolic activity and physiological performance [64–66]. Previous studies corroborate these findings, reporting that spirulina extracts improve vegetative growth, leaf area, biomass, and yield in various crops through enhanced nutrient assimilation and enzyme activation [67, 68]. Seaweed extracts also improved growth and chemical traits compared to the control, aligning with its recognized phyto-stimulatory properties. Seaweed contains polysaccharides (alginates, agar) and phytohormones such as auxins and cytokinins, which promote cell division, photosynthesis, and stress tolerance [69–71]. Its application has been shown to enhance biomass, essential oil content, antioxidant activity, and nutrients in several crops [72–75].

These effects are linked to improved turgor pressure, enhanced respiration, and increased photosynthetic efficiency, ultimately leading to higher productivity.

Collectively, these results highlight the potential of spirulina and seaweed extracts as sustainable biostimulants for improving crop performance under diverse conditions. Their integration into crop management strategies could enhance productivity while reducing reliance on synthetic inputs.

### Impact of the combined effect of organic amendments and phytostimulants on borage performance

The present study demonstrates that the combined application of organic materials and phytostimulant extracts significantly enhances the growth, yield, and biochemical composition of the borage plant. These improvements are attributed to the synergistic effects of organic enrichment and bioactive compounds provided by both amendments. This can provide a balanced nutrient supply and enhance nutrient uptake efficiency, resulting in improved growth and yield [20, 76]. The observed increase in seed yield and nutrient accumulation (N, P, K) under combined treatments suggests a complementary mechanism: organic wastes enhance soil fertility and microbial dynamics. At the same time, phytostimulant extract boosts physiological processes at the foliar level [77, 78]. This dual action likely facilitates more efficient nutrient translocation and assimilation, as evidenced by elevated macronutrient concentrations and improved seed traits [79].

Overall, the integration of SMS and spirulina extract appears to activate both soil-mediated and foliar-mediated growth mechanisms, resulting in improved agronomic performance and biochemical quality. These findings support the use of sustainable, organic inputs to enhance crop productivity and nutritional value, aligning with current trends in eco-friendly agriculture.

## Conclusion

This study demonstrates that integrating organic amendments (compost at 11.9 t ha⁻¹ and spent mushroom substrate at 6.4 t ha⁻¹) with foliar phytostimulants (spirulina or seaweed extracts) significantly improves soil properties, plant growth, flowering, yield, and biochemical composition of borage under low-organic-matter conditions. While phytostimulants alone enhanced vegetative growth and yield traits, they did not alter soil characteristics, highlighting the complementary role of organic amendments in improving soil fertility. Among all treatments, the combination of spent mushroom substrate with spirulina extract produced the most pronounced improvements, suggesting a synergistic effect on nutrient availability and physiological processes. These findings support the use of organic–biostimulant integration as a sustainable strategy to enhance crop productivity in semi-arid soils. Further research should elucidate the underlying biochemical mechanisms and optimize application strategies for broader agricultural adoption.

## Data Availability

Availability of data and materials: Data is provided within the manuscript.
